# Developing mental health research in sub-Saharan Africa: capacity building in the AFFIRM project

**DOI:** 10.1017/gmh.2016.28

**Published:** 2016-12-19

**Authors:** M. Schneider, K. Sorsdahl, R. Mayston, J. Ahrens, D. Chibanda, A. Fekadu, C. Hanlon, S. Holzer, S. Musisi, A. Ofori-Atta, G. Thornicroft, M. Prince, A. Alem, E. Susser, C. Lund

**Affiliations:** 1 Department of Psychiatry and Mental Health, Alan J Flisher Centre for Public Mental Health, University of Cape Town, 46 Sawkins Road, Rondebosch, Cape Town, South Africa; 2 Centre for Global Mental Health, Health Services and Population Research Department, Institute of Psychiatry, King's College London, UK; 3 Department of Mental Health, College of Health Sciences, University of Malawi, Blantyre, Malawi; 4 Department of Psychiatry, College of Health Sciences, University of Zimbabwe, Harare, Zimbabwe; 5 Department of Psychiatry, School of Medicine, College of Health Sciences, Addis Ababa University, Addis Ababa, Ethiopia; 6 Department of Psychiatry, Faculty of Medicine, Makerere University, Kampala, Uganda; 7 Department of Psychiatry, School of Medicine and Dentistry, University of Ghana, Accra, Ghana; 8 Mailman School of Public Health, Columbia University, New York, USA

**Keywords:** AFFIRM, capacity building, low- and middle-income countries, mental health research, sub-Saharan Africa, Teaching and Learning

## Abstract

**Background:**

There remains a large disparity in the quantity, quality and impact of mental health research carried out in sub-Saharan Africa, relative to both the burden and the amount of research carried out in other regions. We lack evidence on the capacity-building activities that are effective in achieving desired aims and appropriate methodologies for evaluating success.

**Methods:**

AFFIRM was an NIMH-funded hub project including a capacity-building program with three components open to participants across six countries: (a) fellowships for an M.Phil. program; (b) funding for Ph.D. students conducting research nested within AFFIRM trials; (c) short courses in specialist research skills. We present findings on progression and outputs from the M.Phil. and Ph.D. programs, self-perceived impact of short courses, qualitative data on student experience, and reflections on experiences and lessons learnt from AFFIRM consortium members.

**Results:**

AFFIRM delivered funded research training opportunities to 25 mental health professionals, 90 researchers and five Ph.D. students across 6 countries over a period of 5 years. A number of challenges were identified and suggestions for improving the capacity-building activities explored.

**Conclusions:**

Having protected time for research is a barrier to carrying out research activities for busy clinicians. Funders could support sustainability of capacity-building initiatives through funds for travel and study leave. Adoption of a train-the-trainers model for specialist skills training and strategies for improving the rigor of evaluation of capacity-building activities should be considered.

## Background

Mental and substance use disorders account for 7.4% of global burden of disease and are among the leading global causes of life lived with disability (Whiteford *et al.* 2014). The proportion of people receiving treatment for mental disorders remains low, particularly in low- and middle-income countries (LMICs), including the sub-Saharan African region (the focus of this paper), where it is estimated to be <10% (Wang *et al.* 2007). Although recent investments in mental health services research in LMICs, such as those made by National Institute of Mental Health (NIMH), are set to make a significant contribution to the evidence base relating to task-shifting, scale-up and integration of mental health treatment and care (Collins *et al.* 2013), there remains a large disparity in the quantity, quality and impact of mental health research carried out in LMICs, relative to both the burden and the amount of research carried out in other regions (Thornicroft *et al.* 2012).

Underlying this deficit are gaps in capacity and inadequate, unevenly distributed resources for research. For example, a recent mapping exercise found that 84% of LMIC mental health publications were written by researchers from 12 countries. LMICs with the highest outputs were often the richest: Brazil, China and India alone accounted for 43% of indexed mental health publications and almost 40% of researchers (Razzouk *et al.* 2010). Only 3% of clinical trials carried out in LMICs were relevant to mental health and most of these were conducted in China (Sheriff *et al*. 2008). Nonetheless, great strides have been made in recent years toward increasing the visibility of mental health within the global health agenda. This has resulted in increased political support, as indicated by the inclusion of mental health in the Sustainable Development Goals (Collins & Saxena, 2016) and the commitment of member states to the WHO Mental Health Action Plan Funding (Saxena *et al*. 2013), and increased funding by the three largest global funders supporting mental health activities (Grand Challenges Canada, UK Department for International Development and US NIMH) (Collins *et al.* 2011). The high-level WHO/World Bank meeting ‘Out of the Shadows: Making Mental Health a Global Priority’ held in Washington DC in April 2016 further attests to this increased recognition for funding.

The historical absence of mental health from the public health agenda has inevitably impacted upon mental health research. Enduring barriers to mental health research in LMICs are underexplored. A number of specific challenges have been identified, including lack of mental health professionals and trained researchers, limited peer networks, insufficient technical expertise, inadequate funding, a limited research culture within universities and lack of opportunities for collaboration (Thornicroft *et al.* 2012). If targets such as those set by the WHO Mental Health Action Plan are to be achieved, research will be necessary in order to track progress, identify obstacles to implementation and find ways in which barriers may be overcome (Collins & Saxena, 2016). Mental health research capacity in sub-Saharan Africa is essential to enabling the indigenization of local mental health practice to reflect the diverse realities of Africa's health systems, socio-economic contexts and cultures, including improving access to appropriate, effective and sustainable services that can address the complex relationship between poverty and mental health (World Health Organization, 2010; Lund *et al.* 2011). Research is also necessary in order to extend psychiatric epidemiology to explore variation within and across countries of Africa, and across regions of the globe, including Africa (Susser & Patel, 2014).

The AFFIRM consortium is one of the five Collaborative Research Hubs in Global Mental Health, supported by the National Institute of Mental Health (NIMH), initiated in 2011 and funded for 5 years (Lund *et al.* 2015). The overall goal of AFFIRM was to improve the delivery and cost-effective mental health interventions in sub-Saharan Africa. This goal was made up of four interconnected aims. Aim one was to generate research on narrowing the treatment gap: trials of task-shared interventions to address maternal mental health in Khayelitsha, South Africa (Lund *et al.* 2014) and management of people living with severe mental illness in primary care in Butajira, Ethiopia are currently underway (Hanlon *et al.* 2016). The second aim was building capacity for research in the sub-Saharan African region. In order to achieve this, the consortium included capacity-building partners from six countries (South Africa, Ethiopia, Ghana, Uganda, Malawi and Zimbabwe). Aims three and four were to promote collaboration: by developing networks across the region and working with other hubs. In general, there is a lack of evidence regarding the types of capacity-building activities that are effective in achieving desired aims and appropriate methodologies for evaluating success. The aim of the AFFIRM consortium was to offer a preliminary, exploratory contribution to this scarce evidence base. We used published literature as well as dicussions among team members (all of whom had experience of carrying out research in low-income settings) as a starting point to identify current and specific challenges. The AFFIRM team were particularly keen to address gaps in capacity of people at different stages in their careers. As described in this paper, capacity building was a cross-cutting activity that was integral to addressing all four AFFIRM aims.

### Aims

1. To provide funded research training and support for mental health and development professionals.
2. To provide brief funded specialist research skills training for people working in research.
3. To provide opportunities for building peer networks, collaboration and career development.
4. To evaluate the content, delivery and impact achieved by the AFFIRM CB program.

### Objectives

- To provide funded places on the M.Phil. program for five health practitioners and managers per year from capacity-building partner countries for the duration of AFFIRM (i.e. 25 AFFIRM fellows in total).
- To provide opportunities for Ph.D. students to carry out projects nested within the AFFIRM hub.
- To undertake a capacity-building needs assessment within the AFFIRM network to determine priority topics for short courses.
- To organize and run a series of short courses addressing priority topics.
- To support the career development of AFFIRM fellows, Ph.D. students and short-course attendees, through building research networks, supporting conference attendance and participation in AFFIRM project meetings.
- To evaluate progress in meeting aims and objectives.

## Methods

### Setting

AFFIRM capacity-building activities build on other mental health capacity development and research initiatives involving a number of LMICs in project consortia, such as the Mental Health and Poverty Project (MHaPP) (Cooper *et al.* 2012), the PRIME project (Program for Improving Mental Health Care) (Lund *et al*. 2012) and the Emerald program (Emerging Mental Health Systems in Low- and Middle-Income Countries) (Semrau *et al.* 2015). The Alan J Flisher Centre for Public Mental Health (CPMH) at the University of Cape Town (UCT) is the lead institution for the AFFIRM, MHaPP and PRIME projects and for a component of the Emerald program. While all of these initiatives included capacity-building, short courses and doctoral opportunities, AFFIRM additionally provided fully-funded fellowships to complete a formal postgraduate qualification at masters level, the M.Phil. in Public Mental Health. The M.Phil. program was led and delivered by the CPMH, a joint UCT and Stellenbosch University (SU) centre. Short courses were designed and delivered by Centre for Global Mental Health, Kings College London (KCL). Ph.D. student projects were nested within the Ethiopian and South African trials and students were supervised by members of the consortium. Capacity-building partners in Ethiopia, Ghana, Uganda, Malawi and Zimbabwe were selected on the basis of having sufficient existing research infrastructure and resources for in-country supervision and support. Researchers from high-income country partner institutions (KCL, Johns Hopkins School of Public Health, Columbia University) were available as potential mentors/supervisors to AFFIRM participants. Further information about the capacity-building program (including open access materials from the short courses) is available via the AFFIRM website (http://www.affirm.uct.ac.za/affirm/components/capacity_building).

### Design of capacity-building activities

#### M.Phil. Public Mental Health

The program, run jointly by UCT and SU, was designed to equip health care practitioners and managers with essential skills to enable them to: plan and evaluate the services that they deliver and manage; communicate and work effectively with national, provincial and district health planners; design and conduct high-quality mental health research; transition from clinical to roles in planning, management, policy, research and leadership. The program was designed to be relevant and accessible to full-time working health and development professionals, building on their existing professional skills and knowledge.

The launch of the M.Phil. program coincided with the start of the AFFIRM project in 2011, which allowed five fellowships to be awarded each year for the 5 years of the project, with a goal of producing 25 M.Phil. graduates by the end of the 5-year program. While the sustainability of the M.Phil. programme was facilitated through the 5 years of AFFIRM fellowships, ongoing sustainability is ensured through self-funded students and sourcing of other funding for further fellowships, such as the AMARI programme described below. The fellowships targeted mental health and development professionals in partner countries other than South Africa (one fellowship per country per year). The criteria for selection of M.Phil. fellows included: completion of a 4-year or honours-equivalent degree in a relevant discipline, previous research experience, coherence and relevance of the proposed research study, academic writing skills, current work context, potential for providing leadership in developing mental health services in their country and recommendations from two referees.

The program consisted of three weeks face-to-face tuition, followed by in-country support and supervision and distance supervision to complete a research project in their home country. Students were expected to submit a dissertation of up to 20 000 words based on their research and complete the M.Phil. within 2 years from registration. Face-to-face training was delivered at the start of the academic year in Cape Town by specialists from UCT and SU. These 3 weeks provided the students with basic skills and knowledge on public mental health, epidemiology, statistics, qualitative methods and analysis, academic writing and systematic reviews, for conducting their own research. At the end of these 3 weeks, the students presented a draft proposal, which was reviewed by their peers and supervisors. Students continued working on their proposals, ethics committee submissions, data collection and writing up of their dissertation in their home countries with support from their supervisors. A small grant of US$1000 was provided to each fellow who received ethical approval for their proposal, to facilitate data collection.

Informal feedback from students suggested ongoing structured academic support was needed in order to support continuation of momentum, successful data collection, write-up and submission. To provide more active and regular support and to develop peer-to-peer networks, webinars were integrated into the M.Phil. curriculum in 2014. This was facilitated by the appointment of an M.Phil. course co-coordinator. Webinars consist of two components: a presentation by the course co-ordinator focusing on a particular technical area or skill relevant to student projects (e.g. how to respond to reviewers, utilizing qualitative software packages), and an opportunity to ask questions and share experiences regarding the research process and receive feedback from the co-ordinator and their peers.

#### Ph.D. program

The two AFFIRM randomized controlled trials (RCTs) offered excellent opportunities for Ph.D. students to carry out high-quality, original research within a rich and supportive learning environment. All Ph.D. students were integrally involved in the trial implementation and selected their topics based on this experience with the benefits of having their fieldwork data collected as part of the trial. Ph.D. students were invited to participate in AFFIRM annual meetings, provided with funding to present their research at conferences, and encouraged to attend AFFIRM short courses. Students were registered on established Ph.D. programs: the Mental Health Epidemiology Ph.D. program at Addis Ababa University (AAU) or the Public Mental Health Division Ph.D. program at UCT.

#### AFFIRM short courses

Topics for short courses covering particular technical skills were identified using findings from the capacity-building needs assessment, carried out among members of capacity-building partner's networks in February 2013. Respondents were asked to rate: (a) their current capacity, (b) the importance of improving their capacity and (c) the level of capacity-building support they felt able to provide for 25 different research-related skills. RCTs and Operational Research (OR) in Public Mental Health were selected as priority topics and designed and delivered as short courses by members of the Centre for Global Mental Health, KCL. Tutors drew upon their experience of other capacity-building activities, including the M.Sc. in Global Mental Health. Short courses combined didactic lectures with short practical exercises and a longer group practical with student presentations of research questions, and study outlines designed in response to course content.

The RCTs in Mental Health short course was held first at UCT (November 2013). Due to positive feedback and a demand for RCT skills training, this course was re-run at AAU in January 2015. The curriculum covered: outline of the RCT design and when it should be used, different types of trial, ethics and good clinical practice, developing research questions, critical appraisal and systematic review, key design elements, analysis and reporting results for achieving impact, and an introduction to OR using mixed methods.

The OR in Public Mental Health in Low-Income Countries short course was held at the University of Malawi College of Medicine in March 2015. The curriculum covered: the chronic disease care model, development of questions for OR, methods and analysis, examples of OR, how to write for peer review journals, dissemination to community and decision-makers, and measuring impact.

### Evaluation of capacity-building activities

Capacity-building activities were evaluated using quantitative and qualitative methods. We examined completion rates and time taken to complete the M.Phil., and asked students to alert us to publications prepared, submitted and published and proposals prepared and submitted. We described data from short course evaluations given to all participants. Participants were asked about their perceptions of quality of each session, whether expectations of the course had been met, and retrospective assessment of change in level of knowledge of the core skills covered in the course. All students who had graduated by the end of 2015 were asked to complete a brief evaluation of their experience of the program, covering, for example, their experiences of the course content and supervisory process, and the impact of the course on their own work and career development. Key themes were identifed from free text answers. The reflections of AFFIRM hub members on progress with capacity-building activities were integral to annual AFFIRM meetings and recorded in meeting minutes. Key reflections and resulting actions are included here.

## Results

### M.Phil. program

Table 1 sets out the professional background of the AFFIRM fellows over the 5 years.

**Table 1.** Professional background of AFFIRM M.Phil. fellows

*N* (across 5 years)	Student background
8	Psychiatrist development worker teaching assistant
4	Psychiatric nurse, Executive Director of NGO
4	Clinical officer (Mental Health)
5	Clinical psychologist
2	Social worker/development worker
1	Executive Director of NGO
1	Teaching assistant

Table 2 describes enrolment and completion of the M.Phil. program by AFFIRM fellows by year of enrolment. In total, 25 AFFIRM funded fellows enrolled. An additional 14 self-funded students enrolled during the AFFIRM project duration. While 40% (10) of the AFFIRM fellows had graduated by June 2016, of the 14 self-funded students, four (28%) had graduated. Majority of the self-funded students were registered only since 2014 and are still in progress.

**Table 2.** Enrolment and completion of M.Phil. in Public Mental Health by AFFIRM fellows

	Number of AFFIRM fellows enrolled (*N*)	Completed by June 2016 (*N*)	Dropped out by June 2016 (*N*)	Died (*N*)	In progress (*N*)
2012	5	4	1	0	0
2013	5	4	0	1	0
2014	5	2	0	0	3
2015	5	0	0	0	5
2016	5	0	0	0	5
Total	25	10	1	1	13

Two graduates (one AFFIRM fellow) have published their thesis (Udedi *et al.* 2013; Benson-Martin & Milligan, 2015). Five graduates have manuscripts in preparation. Two graduates have been involved in the preparation and submission of grant applications, whilst two were enrolled in Ph.D. programs and five more were hoping to embark on a research career via a Ph.D. program. Udedi has secured a position as focal person for mental health within the Ministry of Health, Malawi, since completing the program, and contributed as a tutor to the AFFIRM OR short course.

The individual research topics chosen by the M.Phil. students addressed relevant public mental health areas. This included topics such as epidemiology, instrument development, developing service models and interventions, understanding determinants of mental disorders, and developing policies related to the delivery of mental health services. The research could be qualitative or quantitative and were generally linked to the students’ work context by addressing needs arising and collecting data from these contexts. Given that these students will continue working in these contexts, the impact of undertaking the research should feedback, in the medium- to long-term, into this context given the transferable nature of research skills.

#### Qualitative results from program evaluation carried out among graduates in 2015

Ten of 12 M.Phil. graduates (AFFIRM and self-funded) completed the evaluation (83%). Overall, all of the students felt that after completing the M.Phil. in Public Mental Health they had gained skills in research methods and were well equipped to read a journal article and critically appraise its quality. When considering the 3-week course they attended in Cape Town, seven students thought that the course was long enough and was sufficiently detailed. The remaining three students felt that the course could be improved if the length of this course was extended to allow for consolidation of knowledge. In terms of supervision, all students agreed that their supervisor provided constructive feedback in a timely manner. Supervisors were perceived to be approachable and encouraging, supporting students to push themselves to deliver the highest quality work possible. All four survey respondents who enrolled after 2014 and participated in webinars felt strongly that these supported the timely completion of their thesis and should be permanently integrated into the M.Phil. curriculum. The space to ask questions was appreciated especially as it allowed for immediate answers to be provided.

#### Challenges and benefits of the program raised by AFFIRM members at annual meetings

Supervision from a distance was sometimes problematic especially when students were not easily reachable through email or skype calls. The role of the in-country co-supervisor was crucial in assisting in this communication with students. The addition of monthly webinars from the third intake of students greatly enhanced the ongoing learning process and helped to ensure students kept to the agreed work schedules.

The relationship between UCT/SU supervisors and in-country partners developed iteratively over the course of the project. Initially, in-country partners felt disconnected from selection processes and that their role in supervision was unclear. It was decided that, if one of the AFFIRM countries had more than one suitable applicant for the AFFIRM fellowship, the final decision should be made by the in-country partner. An in-country co-supervisor was identified for each AFFIRM fellow and formally appointed as such. Co-supervision was felt to faciltiate M.Phil. completion and generate post-graduation collaboration in some cases.

Members of the AFFIRM hub raised a number of issues regarding the design and delivery of the M.Phil. program and what it was supposed to achieve. A particular challenge was how to identify students with potential for future leadership. It was felt that these were professionals employed in full-time positions who would find it difficult to meet deadlines and ‘be a student again’. The budget constraints placed on the AFFIRM fellows were challenging, due to costs of data collection and registering for a third year should they not complete in the stipulated 2 years.

### Ph.D.s

Table 3 sets out the topics and completed publications for the five Ph.D. students. In addition to publications, all of the Ph.D. students have participated in annual AFFIRM meetings and received feedback at different stages of their project from consortium members. Four of the Ph.D. students presented their work at the Malawi Mental Health Conference in Blantyre, Malawi in March 2015. One of the Ethiopian students received funding from AAU to complete a 6-week institutional visit to KCL to work with Centre for Global Mental Health researchers on Ph.D. and related AFFIRM activities, which has resulted in the submission of a joint publication.

**Table 3.** AFFIRM Ph.D. students

Student country	Academic background	Title	Date registered	Expected submission date	Ph.D. related publications?
South Africa	Clinical Social Work	Qualitative process evaluation of the basic counseling intervention for depressed pregnant women tested in the South African RCT	2013	2017	(Nyatsanza *et al.* 2016)
South Africa	Registered counsellor	Experiences and measurement of depression and relationships to poverty, food insecurity and social support	2015	2018	(Davies *et al.* 2016)
South Africa	Research Psychologist	Modeling the natural trajectories of maternal depression symptoms in Khayelitsha	2015	2018	
Ethiopia	Clinical psychologist	Functioning in people with severe mental disorders in rural Ethiopia: toward a socio-culturally appropriate instrument development and validation	2013	2017	(Habtamu *et al.* 2015)
Ethiopia	Nursing and Masters in integrated clinical and community mental health	The impact of training and clinical exposure on attitudes of primary health care workers toward mental health care and people with mental illness in Butajira, Ethiopia	2014	2018	

### Short courses

The needs assessment survey was sent out to 40 partners across the six AFFIRM countries, and we received 17 responses (43%). Economic analyses, skills related to dissemination and trials were the top areas with the largest capacity gap. Skills related to intervention design and dissemination made up the top five capacity priorities. Capacity for research was unevenly distributed among sub-Saharan African countries; for example, although capacity needs and gaps tended to be similar to those from other countries, in Malawi, mean scores for current capacity tended to be lower than those from respondents from other sites.

In total, 100 professionals working in the area of mental health in LMICs were reached by these three short courses, as set out in Table 4. There was an increase in retrospectively assessed change in knowledge over time showing we learnt from our experience of the first short course. Participants at AAU seemed to rate their skills lowest at start and report biggest overall improvement. It is difficult to judge why there was this difference and demonstrates a need for standardized measures of skills with demonstrable validity in these settings.

**Table 4.** Participants on the AFFIRM short courses and evaluation outcomes

	RCTs in mental health research		Operations research
	Cape Town 4th–8th November 2013	Addis Ababa 26th–30th January 2015	Blantyre 26th–27th March 2015
Number of participants	27	30	34
Number of feedback forms received (% response rate)	18 (67.0)	22 (73.0)	24 (70.1)
Mean self-rated knowledge after the course (change in score) 1 = very weak 2 = weak 3 = OK but could be improved 4 = strong 5 = very strong			
First steps – Developing research questions, hypotheses, ethics and good clinical practice	3.9 (+0.6)	4.3 (+1.5)	
Key design elements – randomization, sequence generation, allocation, inclusion/exclusion, fidelity	3.8 (+1.1)	4.1 (+1.8)	
Analysis and reporting	3.4 (+0.8)	4.1 (+1.7)	
Dissemination	3.8 (+0.9)	4.5 (+1.6)	
Definition and scope of operational research (OR)			3.8 (+1.5)
Methods and analysis for OR			3.6 (+1.5)
Writing for peer reviewed journals			4.2 (+1.5)
Dissemination to decision-makers and measuring impact			4.1 (+0.7)

## Discussion

Offering funded research training opportunities to 25 mental health professionals, 90 researchers and five Ph.D. students across six countries, the AFFIRM program represents a significant contribution to building research capacity across the sub-Saharan African region. M.Phil. graduates and Ph.D. students have produced a number of high quality publications after their involvement in the program, giving graduates quantifiable outputs and simultaneously contributing to the important growing evidence base of mental health research from sub-Saharan Africa led by local researchers. The M.Phil. program was a successful bridge to research activities for a few mental health professionals who have now enroled or are planning to enrol in doctoral studies. Short courses were well received and led to perceived improvements in core skills. Given the students’ research topics and that they will continue working in these contexts, their skills and the findings from their research have the potential to further impact on public mental health in these contexts going forward. However, this impact would need to be measured in the medium to long term.

A number of specific issues arise from our work regarding the design, delivery and potential impact of longer-term academic research training of graduate-level mental health professionals. The duration of AFFIRM funding over 5 years and the gathering of informal feedback from students and AFFIRM consortium members over this time enabled the program to develop iteratively. The extension of the initial face-to-face training period from 2 to 3 weeks and the addition of the webinars appeared to be helpful modifications. Impact was indicated by the better progression of students who enrolled after these changes. Nonetheless, inherent challenges remain to the delivery and long-term impact of similar programs. Students were released from their work-places to attend face-to-face training. However, consistent with challenges identified by others (Thornicroft *et al.* 2012), once students were back in busy clinical settings, finding time and support for research activities and study was a challenge. This is likely to remain an issue given the targeted group selected onto the M.Phil. program. Future similar programs should pay close attention to how best to support students when they are embedded in their local context, including maximizing local mentorships supported by international collaborators. Secondly, slow internet connection remained a barrier to the successful delivery of the webinars.

Despite evidence to support the positive impact of AFFIRM, there is undoubtedly more work to be done to support the sustainability and longer-term impacts of similar future capacity-building initiatives. A number of possible future directions arise from our findings. For example, the majority of short-course training sessions were designed and delivered by tutors from high-income country partners. Train-the-trainer models have been surprisngly underused in capacity-building initiatives in mental health in both clinical and research skills in LMICs (Liu *et al.* 2016), despite evidence of the effectiveness of this approach from other settings (Ahmed *et al.* 2014) and its potential in supporting sustainability. There are potential lessons for funders from our experience. Clearly, finding time for research activities both during and after the training period is a challenge. Funding for study leave would support further development of research skills, proposals and publications. Linked to this, funding for travel, to facilitate attendance of conferences and meetings, would be essential to the development of nascent peer-to-peer networks and collaborations, helping to promote sustainability of impact through the development of a new generation of African mental health research leaders.

Strategies for increasing the strength of methods for evaluation of research capacity warrant further discussion and debate. Our approach had several limitations. Feedback was completed at the end of short courses, thus change in knowledge was retrospective, subjective and subject to social desirability. This could be improved by using a before-and-after design to measure changes in knowledge, attitudes and competencies. Follow-up time may be insufficient to accurately measure outcomes, particularly publications and proposal submissions. Longer-term impact will be reflected in career advancements, publications with sub-Saharan African lead authors, and successful grant applications from principal investigators from these countries. In order to understand potential barriers to pursuing research activities, it would be informative to carry out research with those who are uninvolved in research after their participation in AFFIRM. It is difficult to quantify the true ‘reach’ of our activities. AFFIRM may have contributed to the development of research ‘culture’ at the universities, governmental and non-governmental organizations and services at which our graduates of short courses and M.Phil. program work. However, this impact remains unmeasured.

Two new initiatives involving consortium members will build directly upon the work of AFFIRM to continue to develop mental health research capacity in sub-Saharan Africa. The first is a networking opportunity – the Mental Health Innovation Network (MHIN)-Africa, initiated under the auspices of the global MHIN initiative (http://mhinnovation.net/organisations/mental-health-innovation-network-africa-mhin-africa) to build networks and channels through which to communicate and disseminate research findings. The AFFIRM capacity-building participants were encouraged to register and prepare contributions to the network. This provides a targeted networking platform for the region. A second is a direct capacity-building initiative the Wellcome Trust-funded Africa Mental Health Research Initiative (AMARI), which focuses almost entirely on capacity building in four countries – Ethiopia, Malawi, Zimbabwe and South Africa. AMARI is led by the University of Zimbabwe and will provide full funding for students registering for the M.Phil. in Public Mental Health at UCT/Stellenbosch University, and doctoral and post-doctoral studies at universities in all four countries. The emphasis is on developing career pathways from masters to Ph.D. and from junior to senior researchers and healthcare leaders.

## Conclusions

Given the increased attention given by donors and governments to mental health, the current roll-out of new services and global and national targets set in order to close the treatment gap, the capacity-building activities of AFFIRM and other research consortia are timely and provide one of the first formal capacity-building initiatives targeting mental health in Sub-Saharan Africa. The need for more research from the region is widely acknowledged and there is some discussion regarding barriers to research as well as recognition of the necessity of capacity building to support this. However, the evidence base on the feasibility, acceptability and effectiveness of capacity-building activities and methods for evaluating these outcomes is weak. In order to support the development of evidence-based capacity-building activities, we have presented detailed information on the aims and objectives of our capacity-building work stream and how these were operationalized. We have presented the results of our evaluation of our activities alongside the reflections of AFFIRM consortium members on their experiences of participating in the delivery of these, in order to stimulate further debate regarding optimum methods for evaluating the impact of similar capacity-building initiatives, including the future work of AMARI and MHIN-Africa.
